# The ACTIVE (Acute Cholecystitis Trial Invasive Versus Endoscopic) study: Multicenter randomized, double-blind, controlled trial of laparoscopic (LC) versus open (LTC) surgery for acute cholecystitis (AC) in adults

**DOI:** 10.1186/1745-6215-9-1

**Published:** 2008-01-10

**Authors:** Fausto Catena, Luca Ansaloni, Salomone Di Saverio, Filippo Gazzotti, Stefano Gagliardi, Federico Coccolini, Luigi D'Alessandro, Giorgio Ercolani, Carlo Talarico, Uberto A Bassi, Leonardo Leone, Filippo Calzolari, Antonio D Pinna

**Affiliations:** 1 General, Emergency and Transplant Surgery DPT, St Orsola-Malpighi University Hospital, Via Massarenti 9. 40138. Bologna, Italy; 2 Italian Society of Young Surgeons (S.P.I.G.C.), Via M Schipa 2, Napoli, Italy

## Abstract

**Background:**

In some randomized trials successful laparoscopic cholecystectomy for cholecystitis is associated with an earlier recovery and shorter hospital stay when compared with open cholecystectomy. Other studies did not confirm these results and showed that the potential advantages of laparoscopic cholecystectomy for cholecystitis can be offset by a high conversion rate to open surgery. Moreover in these studies a similar postoperative programme to optimize recovery comparing laparoscopic and open approaches was not standardized. These studies also do not report all eligible patients and are not double blinded.

**Design:**

The present study project is a prospective, randomized investigation. The study will be performed in the Department of General, Emergency and Transplant Surgery St Orsola-Malpighi University Hospital (Bologna, Italy), a large teaching institutions, with the participation of all surgeons who accept to be involved in (and together with other selected centers). The patients will be divided in two groups: in the first group the patient will be submitted to laparoscopic cholecystectomy within 72 hours after the diagnosis while in the second group will be submitted to laparotomic cholecystectomy within 72 hours after the diagnosis.

**Trial Registration:**

TRIAL REGISTRATION NUMBER ISRCTN27929536 – The ACTIVE (Acute Cholecystitis Trial Invasive Versus Endoscopic) study. A multicentre randomised, double-blind, controlled trial of laparoscopic versus open surgery for acute cholecystitis in adults.

## Introduction

In the developmental stage of laparoscopic cholecystectomy it was considered 'unsafe' or 'technically difficult' to perform laparoscopic cholecystectomy for acute cholecystitis [1,2]. With increasing experience in laparoscopic surgery, a number of centers have reported on the use of laparoscopic cholecystectomy for acute cholecystitis, suggesting that it is technically feasible but at the expense of a high conversion rate, which can be up to 35 per cent [3,4] and common bile duct lesions [5].

Several randomized studies in the early 1980s had shown that performing early open cholecystectomy for acute cholecystitis was better than delayed cholecystectomy in terms of shorter hospital stay but both had similar operative morbidity and mortality rates [3]. Early surgery had since gained in popularity in the late 1980s [2]. Routine use of the open procedure might enable more patients to have the operations during the acute phase because most surgeons are practiced in this approach. The impact of hospital stay and morbidity must also be taken into account. There is the expectation that open operation is associated with more pain and longer hospital stay [6-8].

In some trials successful laparoscopic cholecystectomy during the period of acute inflammation is associated with an earlier recovery and shorter hospital stay when compared with open cholecystectomy [8]. Other studies did not confirm these results and the potential advantages of early laparoscopic cholecystectomy can be offset by a high conversion rate to open surgery [5]. Moreover in these studies a similar postoperative programme to optimize recovery comparing laparoscopic and open approaches was not standardized. Many studies also do not report all eligible patients and are not double blinded.

## Methods

### Design

The study project is a prospective, randomized investigation. The study will be performed in the Department of Emergency Surgery St Orsola-Malpighi University Hospital (Bologna, Italy), a large teaching institutions, with the participation of all surgeons who accept to be involved in (and together with other selected centers).

The patients will be divided in two groups: in the first group the patient will be submitted to early LC (Laparoscopic Cholecystectomy) within 72 hours after the diagnosis of cholecystitis while in the second group will be submitted to early LTC (LaparoTomic Cholecystectomy) within 72 hours after the diagnosis.

#### Randomization

The randomization will be obtained through computer-generated schedule. The result of this randomization will be sealed in numbered envelopes. After cholecystitis diagnosis if the patient fulfils the inclusion criteria the responsible surgeon will ask the patient to partecipate to the study. If the patient agree, he/she will sign the informed consent. After patient's consent the randomization will be carried out. The responsible surgeon will record the patient name (and number). All eligible patients will be recorded.

### Statistics

Sample size has been calculated to reach a confidence level of 95% with a power of 80%.

A sample size of 144 patients is calculated supposing that the hospital stay for LC is shorter than 2 days. The sample size will be 72 patients for each group (144 patients for the whole study).

For comparison of the two groups, chi-square analysis and Fisher's exact test are used when appropriate for qualitative data, and the Student t-test (for normal variables) or the Mann Whitney U-test (for nonnormal variables) for quantitative data. For multivariate analysis the stepwise logistic regression is applied. A probability of 0.05 or less is accepted as statistically significant.

### Inclusion And Exclusion Criteria

Inclusion criteria are:

Adult patients (>18 years)

Clinical (pain, fever > 37.5°C, WBC > 10.000/microL), and ultrasound evidence of cholecystitis

ASA I-III patients

Informed consent

Less than 72 h from the onset

Exclusion criteria

Informed consent refusal

Choledocholithiasis

Generalized peritonitis

Previous abdominal surgical procedures

Patients with an intra-operative findings of different pathology will be excluded from the study

Apache II score > 10

#### Intervention

Preoperative data collected will include patient demographics and comorbid conditions (genitourinary, cardiac, pulmonary, gastrointestinal, renal, or rheumatologic) and a detailed history of symptom onset.

The procedure was performed by a surgeon that had performed at least 50 LCs.

On admission, the patients were started on cefotaxime, 2 g IV every 12 h, which was continued postoperatively according to NNISS score [9].

The standard four-trocar operative technique is used for LC for acute cholecystitis.

When the gallbladder is distended it will be first aspirated. To allow a good hold on the gallbladder larger graspers will be inserted through a 5 mm right lower port. The cystic artery and duct are clip-ligated. The gallbladder and intraperitoneal "dropped" stones are collected in an endoscopic bag and extracted through the umbilical cannula site, which can be extended. A closed system suction drain is left. Fascial closure is attempted only at the umbilical cannula site. The skin at all the cannula sites are closed with staples.

Laparotomic procedure is carried out with an about 8 cm right subcostal incision and the traditional surgical technique with a closed system suction drain left in situ.

#### Data Collection

Patients' data sheets are generated containing demographic data and preoperative, operative, and postoperative information.

Pre-operative notes concern the history of gallbladder stones, the presence of associated diseases (cardiac, hypertension, diabetes, malignancy), duration of gallbladder complaints (as an indication for the onset of the disease), finding of a palpable gallbladder, temperature, and laboratory results of WBC count, serum bilirubin, gamma GT, PCR, IL-6 and alkaline phosphatase. Ultrasound findings are also reported.

Operative data of concern are macroscopic findings (of acute cholecystitis, gangrenous cholecystitis, hydrops, and empyema of the gallbladder), the presence of small stones (< 1 cm diameter) or large bile stones (> 1 cm diameter), information regarding perforation of the gallbladder and intraperitoneally "lost" stones, reasons for conversion, and duration of surgery. Postoperative notes of interest included the use of nasogastric tubes and drains, the amount of analgesics used, (evaluation of pain with VAS score), complications, and length of hospital stay.

Complications are classified as surgical infections (wound infection, subphrenic or subhepatic abscess); noninfectious surgical problems (e.g., bile duct injury, hemorrhage); remote infections (urinary or respiratory); and miscellaneous problems (e.g., atelectasis, deep vein thrombosis, AMI, CVA, etc). The collected information are entered into a database as either continuous or categorical variables for statistical analysis. Following the operative procedure, a large sterile dressing will be applied to cover the entire abdomen.

A second surgical team, aware of the operative findings but not the surgical access approach, then will assume the care of the patient. Postoperative care and ability to be discharged from the hospital will be determined by the second surgical team. This second surgical team will be blinded to the surgical approach. The primary operative team will be in every moment available for emergent consultation or evaluation of the wound.

In the pre-anaesthetic holding area, baseline pain will be investigated at rest and on coughing using a pain-rating scale systems: a 100-mm visual analogue scales (VAS) (0 = minimal and 100 = maximal).

An investigator blinded to the study operation performed, will evaluate postoperative pain intensity on returning to the Surgical Ward (zero time), and after 12 and 24 hours (± 3 hours). The three hours of tolerance (before or after the precise time of control) will be used in order to avoid to awake the patients during the night. But during the day time, if asleep, the patient will be awakened for the pain test. At these timed intervals, the following variable will be recorded: VAS for pain at rest, and on coughing; if, at rest, VAS is more than 3, the patient will be given intravenous 30 mg ketorolac. The pain tests will be also performed whenever the patient ask for additional analgesia between the timed controls and parenteral analgesics will be administered accordingly. Every time the patient undergoes the pain test, he will be asked about the location of pain (in the surgical wound, far from the wound, everywhere or does not know). As soon as patients will begin to drink fluid instead of ketorolac 30 mg iv, they will be offered nimesulide 100 mg 1 tab orally prn (max 2 tabs daily). Orally administered analgesics will be continued in preference to parenterally administered analgesics if they will be efficacious. After discharge, the patients will be offered nimesulide 100 mg 1 tab orally prn (max 2 tabs daily). Also parenteral and oral analgesic drug requirements will be recorded and analyzed as a measure of postoperative pain. Furthermore the patient satisfaction with the analgesia provided (using a scale of poor, satisfactory, good, or excellent) will be recorded before discharge and after 7 days.

Patient discharge will be based on good medical practice criteria: 1) apyrexia 2) absence of diseases requiring hospitalisation 3) return of bowel function 4) patient's compliance.

#### Informed Consent Form Or Information Sheet

In the informed consent form, patients will receive all the information about the study protocol, the confidential nature of personal data and will fill up a questionnaire before signing or refuse. There will be not inconveniences caused to the patients. No incentives are planned for the patients regarding the operation or the follow-up. All the medical informations obtained from the patients will be kept confidentially among the research scientists conducting the study. The patients will be free to withdraw from the study, whenever they want without any obligation.

### Ethical Approval

Approved by the ethical Committee of the Saint Orsola-Malpighi Hospital (see Additional files 1 and 2).

### Primary Endpoints

The aim of the study is to compare the results of early laparoscopic and laparotomic cholecystectomy for acute cholecystitis in terms of morbidity, mortality, conversion rate, operation time, hospital stay, postoperative pain, return to normal activity and aesthetic result.

The primary endpoints of our study will be:

To evaluate the value of laparoscopic cholecystectomy to reduce hospital stay

To evaluate the value of laparoscopic cholecystectomy to reduce postoperative pain

To evaluate the conversion rate

The onset of any other complications will be recorded intraoperatively, postoperatively, at discharge, at 7-days, 1-month and 6-months.

All the above mentioned data will be recorded in the Case Report Form and later stored in computer database. At the end of the study the final statistical examination will be carried out.

An interim statistical examination of the data will be done every 3 months during the period of patients' inclusion in the study. Then at the end of every completed follow-up period (1-month, 6-months).

The statistical analysis will be carried out using Epi Info 2000, Version 1.1 software package (Dean AG, Arner TG, Sangam S, Sunki GG, Friedman R, Lantinga M, Zubieta JC, Sullivan KM, Smith DC. Epi Info 2000, a database and statistics program for public health professionals for use on Windows 95, 98, NT, and 2000 computers; Centers for Disease Control and Prevention, Atlanta, Georgia, USA, 2000)

No incentives are planned for the patients regarding the operation or the follow-up.

The study will take approximately 6 months – 1 year for the inclusion period. According to the number of AC menaged monthly in all Centers, the duration of the inclusion period can be approximately of 1 year to reach the number of about 144 enrolled patients.

An interim report is planned at the end of any completed follow-up period.

AC is a common disease. Any improvement in this field will benefit many patients reducing morbidity, mortality, conversion rate, operation time, hospital stay, postoperative pain, return to normal activity and aesthetic result. All our patients will be informed about the study and an informed consent will be obtained. There will not be inconveniences caused to the patients. All the medical informations obtained from the patients will be kept confidentially among the research scientists conducting the study. The patients will be free to withdrawn from the study, whenever they want without any obligation.

## Competing interests

Each author should have participated sufficiently in the work to take public responsibility for appropriate portions of the content.

All authors read and approved the final manuscript and declare no competing interests.

## Authors' contributions

FC, FG, SD, CT have made substantial contributions to conception and design, or acquisition of data, or analysis and interpretation of data;

LA, SG, GE, AL have been involved in drafting the manuscript or revising it critically for important intellectual content

LL, CT, UAB, Filippo Calzolari participated in the design of the study and performed the statistical analysis.

ADP, LD, Federico Coccolini conceived of the study, and participated in its design and coordination and helped to draft the manuscript.

## Supplementary Material

Additional file 1 Ethical Committee approval. First page of the Ethical Committee approval document. Click here for file

Additional file 2 Ethical Committee approval. Second page of the Ethical Committee approval document. Click here for file
