# Supplementary figures and images for: The ACTIVE (Acute Cholecystitis Trial Invasive Versus Endoscopic) study: Multicenter randomized, double-blind, controlled trial of laparoscopic (LC) versus open (LTC) surgery for acute cholecystitis (AC) in adults
Source: Trials. 2008 Jan 10;9:1. doi: 10.1186/1745-6215-9-1 (PMC2244597; doi:10.1186/1745-6215-9-1)

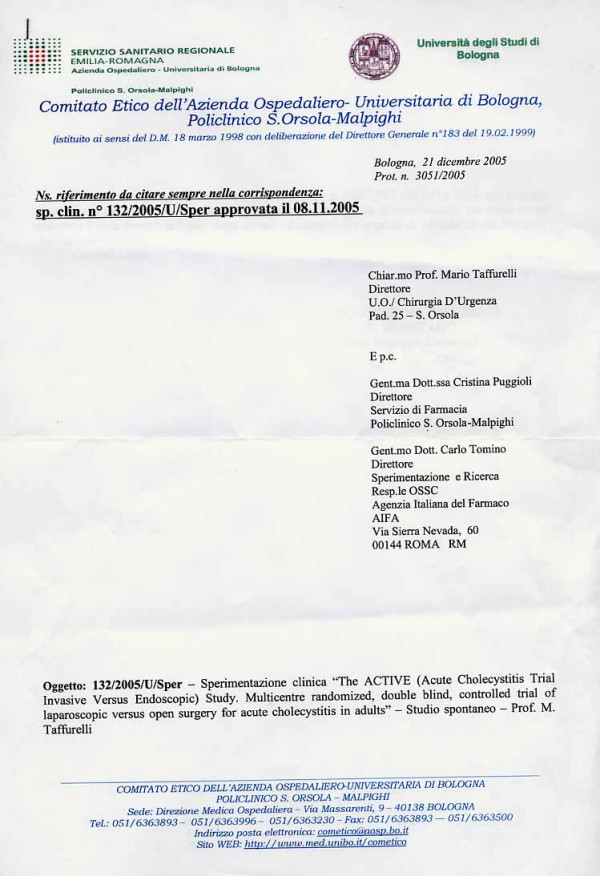

Supplement: Additional file 1 — Ethical Committee approval. First page of the Ethical Committee approval document. [file 1745-6215-9-1-S1.jpeg]

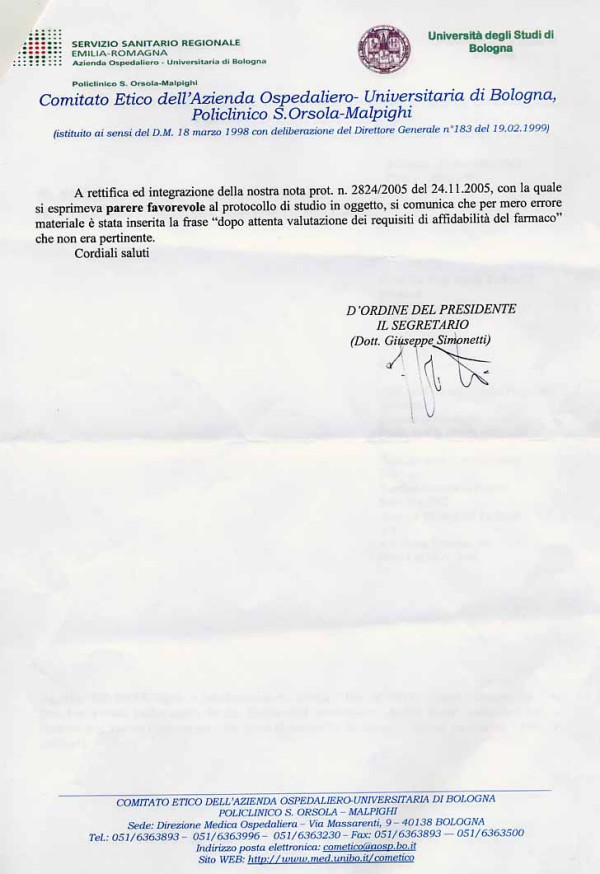

Supplement: Additional file 2 — Ethical Committee approval. Second page of the Ethical Committee approval document. [file 1745-6215-9-1-S2.jpeg]
